# Immunosuppression with Antitumour Necrosis Factor Therapy Leading to *Strongyloides* Hyperinfection Syndrome

**DOI:** 10.1155/2018/6341680

**Published:** 2018-05-30

**Authors:** Muhammad Farhan Khaliq, Rayan E. Ihle, James Perry

**Affiliations:** ^1^Department of Internal Medicine, Charleston Area Medical Center, Charleston, WV, USA; ^2^Department of Pulmonary Critical Care, West Virginia University Charleston Division, Charleston, WV, USA; ^3^Department of Pulmonary Critical Care, Charleston Area Medical Center, Charleston, WV, USA

## Abstract

*Strongyloides stercoralis* is an endemic parasitic infection that can remain asymptomatic for years, but it can cause death in immunosuppressed individuals. Here, we present a case of *Strongyloides* hyperinfection in a 75-year-old male secondary to sepsis and chronic immunosuppression due to TNF-*α* inhibitors. Despite aggressive treatment including broad-spectrum antibiotics and antihelminths, his respiratory failure worsened and he died after palliative extubation. *S. stercoralis* infection remains a diagnostic challenge. Presentation with *Strongyloides* is often nonspecific, and eosinophilia is absent in hyperinfection. Diagnosis can be delayed, especially in low-prevalence areas where suspicion is low. *Strongyloides* should be considered in the differential diagnosis in the presence of risk factors including immunosuppressive therapy, and a travel history should be carefully obtained. Patients with recurrent enterobacterial sepsis or respiratory failure with diffuse infiltrates in the setting of eosinophilia should undergo testing for *Strongyloides*. A multidisciplinary approach can result in earlier diagnosis and favorable outcomes.

## 1. Introduction

*Strongyloides stercoralis* is an intestinal nematode that is endemic in tropical and subtropical areas and affects 370 million people globally [1]. Its presentation can vary from asymptomatic eosinophilia in immunocompetent patients to hyperinfection syndrome causing multiple organ failure in immunocompromised patients. A weakened host immune response results in an increased parasitic load, including in the lungs, referred to as hyperinfection. Larvae migrating beyond the lungs, for example, to the brain or skin, are termed “disseminated *Strongyloides*.” Chronic glucocorticoid therapy, malnutrition, alcoholism, and underlying human T-lymphotrophic virus type 1 (HTLV-1) infection are known risk factors. Despite therapy, prognosis remains poor, with mortality reaching 70% [2, 3].

## 2. Case Report

A 75-year-old male was transferred to our facility due to acute respiratory failure and sepsis secondary to extended-spectrum beta-lactamase *Escherichia coli* urinary tract infection (ESBL-UTI). His past medical history included a recent diagnosis of deep vein thrombosis, after which he developed gastrointestinal bleeding while on anticoagulation therapy. He had rheumatoid arthritis, gout, diabetes mellitus, hypertension, atrial fibrillation, stage III chronic kidney disease, IgM monoclonal gammopathy of undetermined significance, and chronic pain syndrome. His surgical history included placement of an inferior vena caval filter and recurrent bilateral pleural effusions requiring decortication. He recently had a gout flare for which he was treated with a prednisolone taper. The patient's rheumatoid arthritis was controlled with infliximab and hydroxychloroquine sulfate. He was a former smoker but had no significant history of alcohol or illicit drug use. He was from rural West Virginia which is located in the north central subregion of Appalachia. He denied travel outside of the United States.

He originally presented with decreased appetite, nausea, vomiting, and abdominal pain at an outlying facility. At this time, vital signs were normal with a temperature of 36.9°C, blood pressure of 131/68 mmHg, a regular pulse rate of 96 beats/minute, and a respiratory rate of 17. He was alert, awake, and oriented to time and person. The remainder of his physical examination was unremarkable. Initial blood investigations revealed a haemoglobin level of 8.5 g/dL, total white cell count of 8 × 10^9^/L without eosinophilia, and a normal platelet count of 203 × 10^9^/L. Biochemically, there was evidence of impaired renal function with a creatinine level of 2.8 mg/dL, hyponatraemia (Na 122 mEq/L), and hypochloraemia (Cl 92 mEql/L). Liver function tests were unremarkable including his coagulation profile. The patient had *Clostridium difficile* testing, which was negative. An abdominal computed tomography (CT) scan demonstrated only fecal retention. His electrolyte abnormalities were attributed to dehydration from vomiting, so a nasogastric tube was placed while fluid resuscitation was administered. A diagnosis of diabetic gastropathy was made and metoclopramide started. His hospital course was complicated by a UTI secondary to *E. coli* which was resistant to multiple antibiotics. He was treated with ciprofloxacin. However, the patient continued to worsen, and he developed sepsis and respiratory failure requiring intubation and transfer to our facility for higher care.

**Figure 1:**
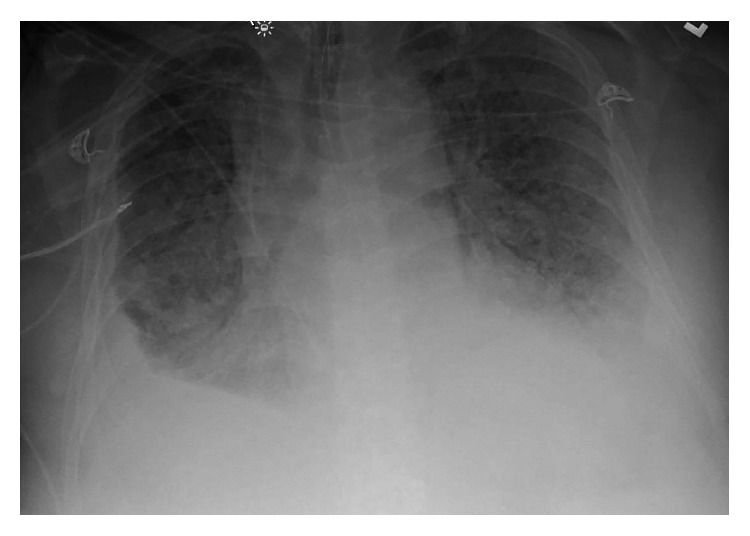
Chest radiograph showing bilateral diffuse infiltrates.

**Figure 2:**
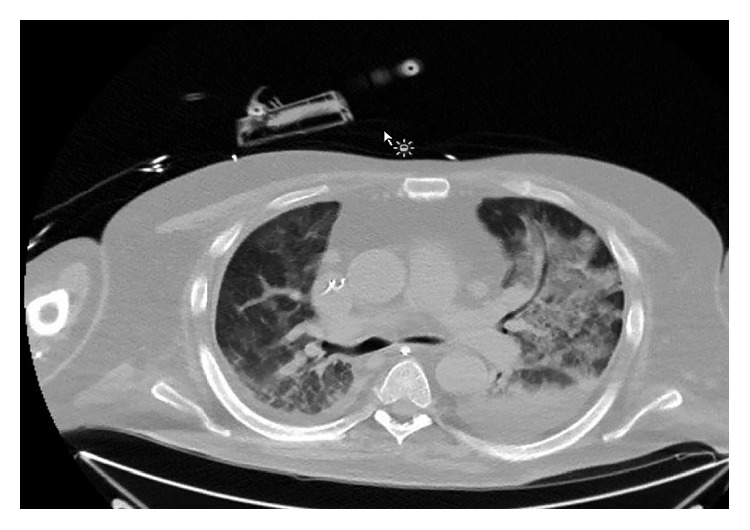
CT scan of the chest showing multifocal airspace disease concerning pneumonia or oedema.

**Figure 3:**
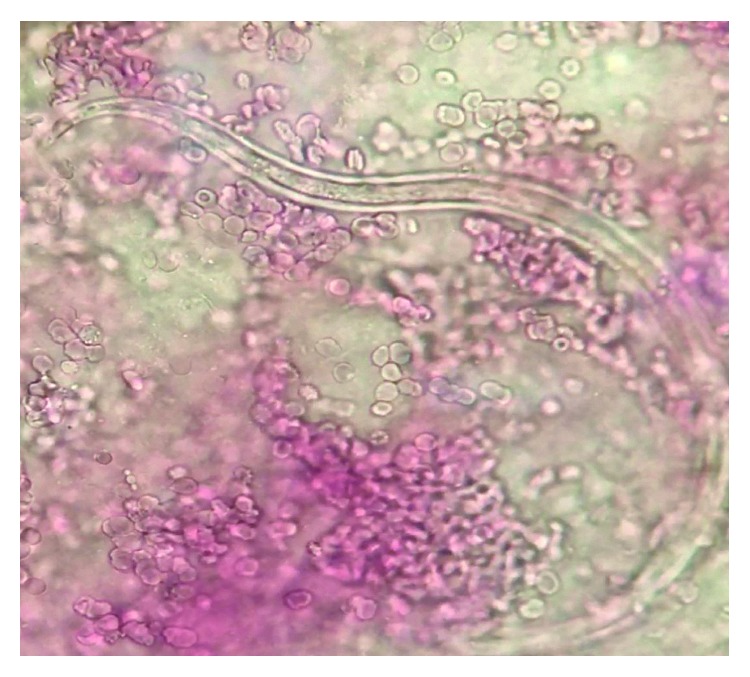
*Strongyloides stercoralis* parasite present in the BALF.

At the time of presentation to our facility, his temperature was 36°C, blood pressure was 101/60 mmHg, pulse rate was 111 beats/minute and regular, and his respiratory rate was 18. His haemoglobin level was 9.0 g/dL, total white cell count was 11.8 × 10^9^/L with eosinophilia of 5%, and platelet count was 174 × 10^9^/L. His procalcitonin level was 0.75 ng/mL. His electrolytes were similar to the outlying facility with low albumin. His troponins were negative, and brain natriuretic peptide was 51 pg/mL. Vasopressors and meropenem were initiated to control sepsis. Stress-dose intravenous hydrocortisone was added for refractory shock and possible adrenal insufficiency due to his recent steroid exposure. A few days later, he suffered from atrial fibrillation with a rapid ventricular rate that required cardioversion and an amiodarone drip. Despite aggressive diuresis at that time, the patient's hypoxia worsened with no improvement on chest radiographs (Figure 1). A CT scan of his chest showed multifocal bilateral airspace disease concerning for pneumonia or oedema (Figure 2). The patient's respiratory and blood cultures remained negative throughout the hospital course. At that time, bronchoscopy was performed to evaluate his nonresolving infiltrates and respiratory failure. Bronchoscopy showed diffuse alveolar haemorrhage. The patient was started on high-dose methylprednisolone 1 g/day for 3 days. His bronchoalveolar lavage fluid (BAL) grew *S. stercoralis* (Figure 3). At that time, ivermectin was added to his regimen. Unfortunately, the patient further deteriorated within 48 hours after diagnosis. The patient's family requested initiation of comfort care, and the patient underwent palliative extubation and died a few hours later.

## 3. Discussion

*S. stercoralis* is an intestinal nematode that is widely distributed throughout the tropics and subtropics [4–6]. In the United States, the majority of cases are seen in migrants and travellers from endemic areas. After initial exposure, infestation with *Strongyloides* can persist for many years. Cases have been reported of *Strongyloides* infection diagnosed 75 years after travel or migration [7, 8]. *Strongyloides* is also known to be endemic to the Appalachian region of the United States to which the entire state of West Virginia belongs [9, 10].

The clinical presentation of *Strongyloides* can vary and depends upon various factors including acuity of infection, host immunity, and organism load. It can present as acute infection, autoinfection, chronic infection, hyperinfection, and disseminated disease. Over half of infected patients are asymptomatic carriers, with eosinophilia being the only laboratory abnormality. Failure to clear the infection can lead to chronic infection characterized by a low parasite load that maintains survival in the host via a well-regulated autoinfection cycle. Immunosuppressive states induced by corticosteroids and TNF-*α* inhibitors or conditions such as lymphoma, HTLV infection, bone marrow allografts, rheumatoid arthritis, alcoholism, malnutrition, leprosy, tuberculosis, and chemotherapeutic drugs can lead to disseminated hyperinfection syndrome [11–14]. TNF-*α* inhibitors along with glucocorticoids can modify the Th2 response which has a role in controlling many helminthic infestations resulting in exacerbation of asymptomatic carrier state to symptomatic infection or hyperinfection [12, 13]. Hyperinfection syndrome is characterized by an increase in the larval load and diffuse infiltration of the organs by the larva causing a systemic inflammatory response- (SIRS-) like condition with multiple organ failure. Often, disruption of the intestinal mucosa and overwhelming acceleration of the autoinfection cycle can result in leaking of enteric bacteria into bloodstream to cause sepsis or meningitis.

*Strongyloides* is difficult to diagnose due to nonspecific symptoms. Infestation of the respiratory or gastrointestinal tracts can present as dyspnoea, cough, wheezing, abdominal pain, diarrhoea, vomiting, ileus, small bowel obstruction, or protein-losing enteropathy. Occasionally, it can give rise to a distinctive form of cutaneous larva migrans, recognized as larva currens. Standard stool examination can be insensitive, as the larvae are shed intermittently and can fail to detect larva in 70% of cases [15]. Multiple stool examinations are required to increase yield if clinical suspicion is high. In hyperinfection syndrome, the larval load significantly increases and becomes detectable in sputum, bronchoalveolar lavage (BAL), and lung biopsies. In our case, larvae were detected in the BAL. Stool samples were not obtained because of the low suspicion and lack of gastrointestinal symptoms. If a high index of suspicion is present with negative stools, serology can be performed with sensitivity and specificity ranging over 95% by various serologic methods [16].

Eosinophilia is the most common isolated finding in asymptomatic carriers but is absent in hyperinfection syndrome. Our patient had eosinophilia during previous hospitalizations and on the initial labwork at the time of admission but was absent otherwise. With worsening sepsis and steroids for gout, neutrophilia could have masked underlying eosinophilia. It is important to note that the presence of eosinopenia indicates a poor prognosis, attributable to the fact that eosinophils are the primary cells that provide immunity against parasitic infections [17].

Our patient had bilateral lung infiltrates with small effusions, consistent with previous cases involving the lungs. *Strongyloides* can present with a completely normal chest radiograph or with abnormalities such as pulmonary infiltrates, lung abscesses, pleural effusions, and interstitial nodular patterns [18]. On bronchoscopy, alveolar haemorrhage and petechial haemorrhages with hyperaemia of the respiratory tract mucosa have also been reported [19].

*Strongyloides* hyperinfection syndrome is a difficult condition to treat and is associated with high mortality [2, 3]. This high mortality is attributable to various factors. First, the diffuse infiltration of multiple organs causes a SIRS-like condition. Secondly, the organism penetrates gastrointestinal and respiratory mucosae, leading to entry of new microbes and therefore secondary infections. Third, a delay in diagnosis in low-prevalence areas can lead to detrimental consequences. Lastly, immunosuppressed states can lead to more severe primary infection consequences as well as secondary infections.

Recently published guidelines in nonendemic areas suggest that immunocompromised patients or patients planning on starting immunosuppressant therapy should be screened if they have high-to-intermediate exposure risk [2]. Serologic testing with ELISA is preferred; however, direct fecal testing can be considered if serology is not available [20]. In case where serology or fecal testing is not available, empiric antihelminthic treatment should be considered prior to initiating immunosuppressant therapy [2, 19]. Ivermectin remains the first choice, and most studies have shown it to be better than albendazole and thiabendazole [21]. It can be used as a single dose or in two doses given consecutive days. Posttreatment confirmation of eradication should be performed with the most sensitive available techniques. Serology at baseline with follow-up at 6 and 12 months after treatment is recommended to monitor the response in titers [20].

## 4. Conclusion

Serious complications including death may occur in patients with chronic *Strongyloides* infection treated with immunosuppressant medications including TNF-*α* inhibitors and corticosteroids. *Strongyloides* hyperinfection usually presents as acute respiratory failure and may initially mimic an asthma exacerbation or infiltrates consistent with bacterial pneumonia.

## Additional Points

*Learning points*. (i) A travel history should be carefully obtained, as the majority of patients in the US are immigrants or travellers from tropical countries. (ii) Suspicion of *Strongyloides* should be high, especially in patients who are immunosuppressed. (iii) In such cases, systemic corticosteroid therapy should be avoided until the diagnosis of *Strongyloides* is definitively ruled out. (iv) If testing is not available, empiric treatment should be considered in patients starting immunosuppressant medications.

## References

[1] Bisoffi Z., Buonfrate D., Montresor A. (2013). *Strongyloides stercoralis*: a plea for action. *PLoS Neglected Tropical Diseases*.

[2] Buonfrate D., Requena-Mendez A., Angheben A. (2013). Severe strongyloidiasis: a systematic review of case reports. *BMC Infectious Diseases*.

[3] Lim S., Katz K., Krajden S., Fuksa M., Keystone J. S., Kain K. C. (2004). Complicated and fatal *Strongyloides* infection in Canadians: risk factors, diagnosis and management. *Canadian Medical Association Journal*.

[4] Montes M., Sawhney C., Barros N. (2010). *Strongyloides stercoralis*: there but not seen. *Current Opinion in Infectious Diseases*.

[5] Hong I. S., Zaidi S. Y., McEvoy P., Neafie R. C. (2004). Diagnosis of *Strongyloides stercoralis* in a peritoneal effusion from an HIV-seropositive man. *Acta Cytologica*.

[6] Kakati B., Dang S., Heif M., Caradine K., McKnight W., Aduli F. (2011). *Strongyloides* duodenitis: case report and review of literature. *Journal of the National Medical Association*.

[7] Prendki V., Fenaux P., Durand R., Thellier M., Bouchaud O. (2011). Strongyloidiasis in man 75 years after initial exposure. *Emerging Infectious Diseases*.

[8] Nabha L., Krishnan S., Ramanathan R. (2012). Prevalence of *Strongyloides stercoralis* in an urban US AIDS cohort. *Pathogens and Global Health*.

[9] Genta R. M. (1989). Global prevalence of strongyloidiasis: critical review with epidemiologic insights into the prevention of disseminated disease. *Reviews Infectious Diseases*.

[10] Berk S. L., Verghese A., Alvarez S., Hall K., Smith B. (1987). Clinical and epidemiologic features of strongyloidiasis: a prospective study in rural Tennessee. *Archives of Internal Medicine*.

[11] Basile A., Simzar S., Bentow J. (2010). Disseminated *Strongyloides stercoralis*: hyperinfection during medical immunosuppression. *Journal of the American Academy of Dermatology*.

[12] Keiser P. B., Nutman T. B. (2004). *Strongyloides stercoralis* in the immunocompromised population. *Clinical Microbiology Reviews*.

[13] Schär F., Trostdorf U., Giardina F. (2013). *Strongyloides stercoralis*: global distribution and risk factors. *PLoS Neglected Tropical Diseases*.

[14] Boatright M. D., Wang B. W. (2005). Clinical infection with *Strongyloides sterocoralis* following etanercept use for rheumatoid arthritis. *Arthritis & Rheumatism*.

[15] Ericsson C. D., Steffen R., Siddiqui A. A., Berk S. L. (2001). Diagnosis of *Strongyloides stercoralis* infection. *Clinical Infectious Diseases*.

[16] Bisoffi Z., Buonfrate D., Sequi M. (2014). Diagnostic accuracy of five serologic tests for *Strongyloides stercoralis* infection. *PLoS Neglected Tropical Diseases*.

[17] Gyorkos T. W., Genta R. M., Viens P., Maclean J. D. (1990). Seroepidemiology of *Strongyloides* infection in the Southeast Asian refugee population in Canada. *American Journal of Epidemiology*.

[18] Vijayan V. K. (2009). Parasitic lung infections. *Current Opinion in Pulmonary Medicine*.

[19] Yee A., Boylen C. T., Noguchi T., Klatt E. C., Sharma O. P. (1987). Fatal *Strongyloides stercoralis* infection in a patient receiving corticosteroids. *Western Journal of Medicine*.

[20] Requena-Méndez A., Buonfrate D., Gomez-Junyent J., Zammarchi L., Bisoffi Z., Muñoz J. (2017). Evidence-based guidelines for screening and management of strongyloidiasis in non-endemic countries. *American Journal of Tropical Medicine and Hygiene*.

[21] Henriquez-Camacho C. A. J., Gotuzzo E., Echevarria J., Terashima A. (2016). Ivermectin versus albendazole or thiabendazole for *Strongyloides stercoralis* infection. *Cochrane Database of Systematic Reviews*.

